# Supplementary material for: Spatio-Temporal Patterns of the SARS-CoV-2 Epidemic in Germany
Source: Entropy (Basel). 2023 Jul 29;25(8):1137. doi: 10.3390/e25081137 (PMC10453630; doi:10.3390/e25081137)
Supplement: Supplementary file 1 [file entropy-25-01137-s001.zip › entropy-2490902-supplementary.pdf]

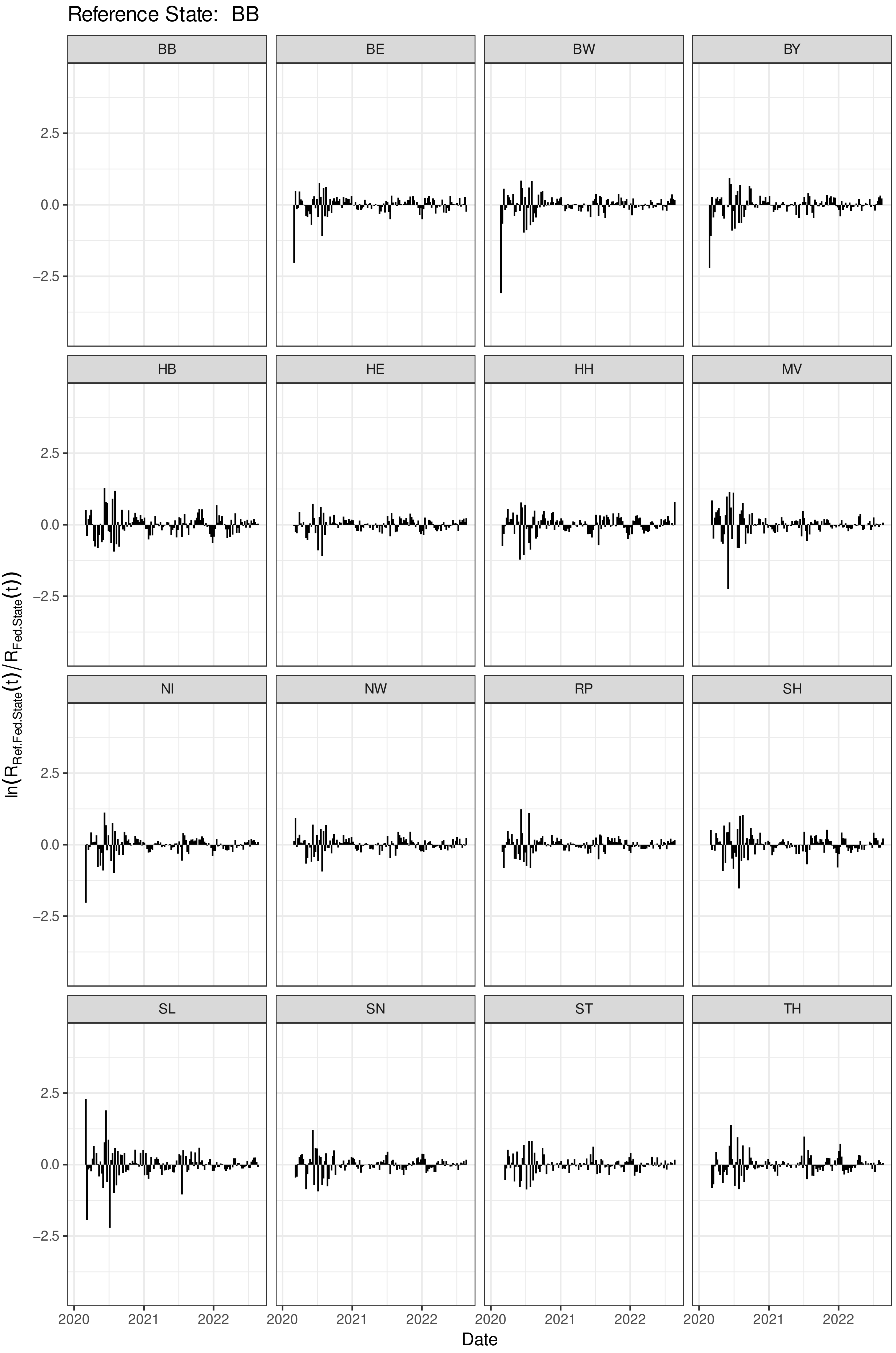
Reference State: BB
BB
BE
BW
BY
HB
HE
HH
MV
NI
NW
RP
SH
SL
SN
ST
TH
2.5
0.0
−2.5
2020
2021
2022
Date
$\ln\left(R_{Ref.Fed.State}(t)/R_{Fed.State}(t)\right)$

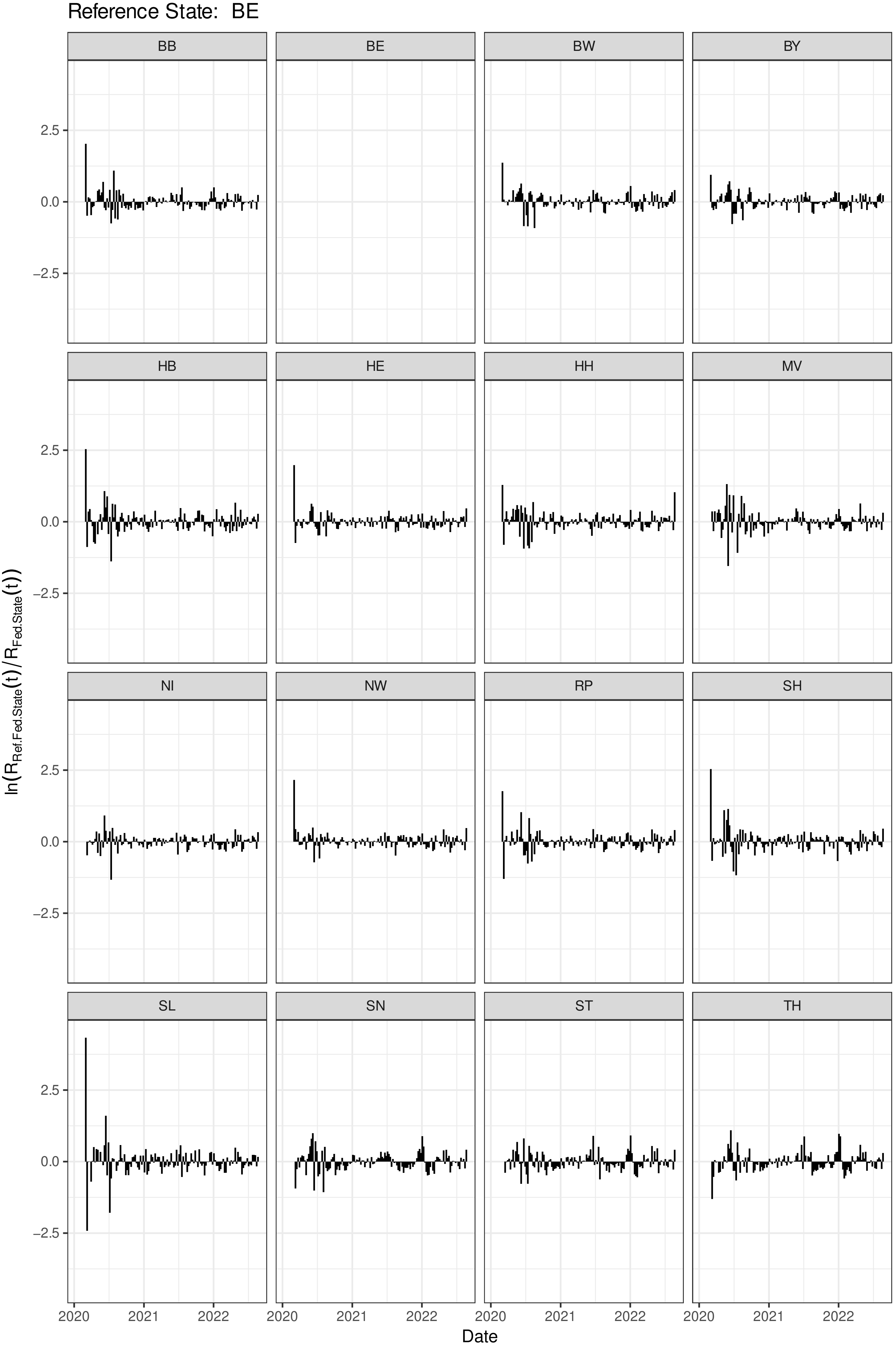
Reference State: BE
BB
BE
BW
BY
HB
HE
HH
MV
NI
NW
RP
SH
SL
SN
ST
TH
2.5
0.0
−2.5
2020
2021
2022
Date
$\ln(R_{Ref.Fed.State}(t)/R_{Fed.State}(t))$

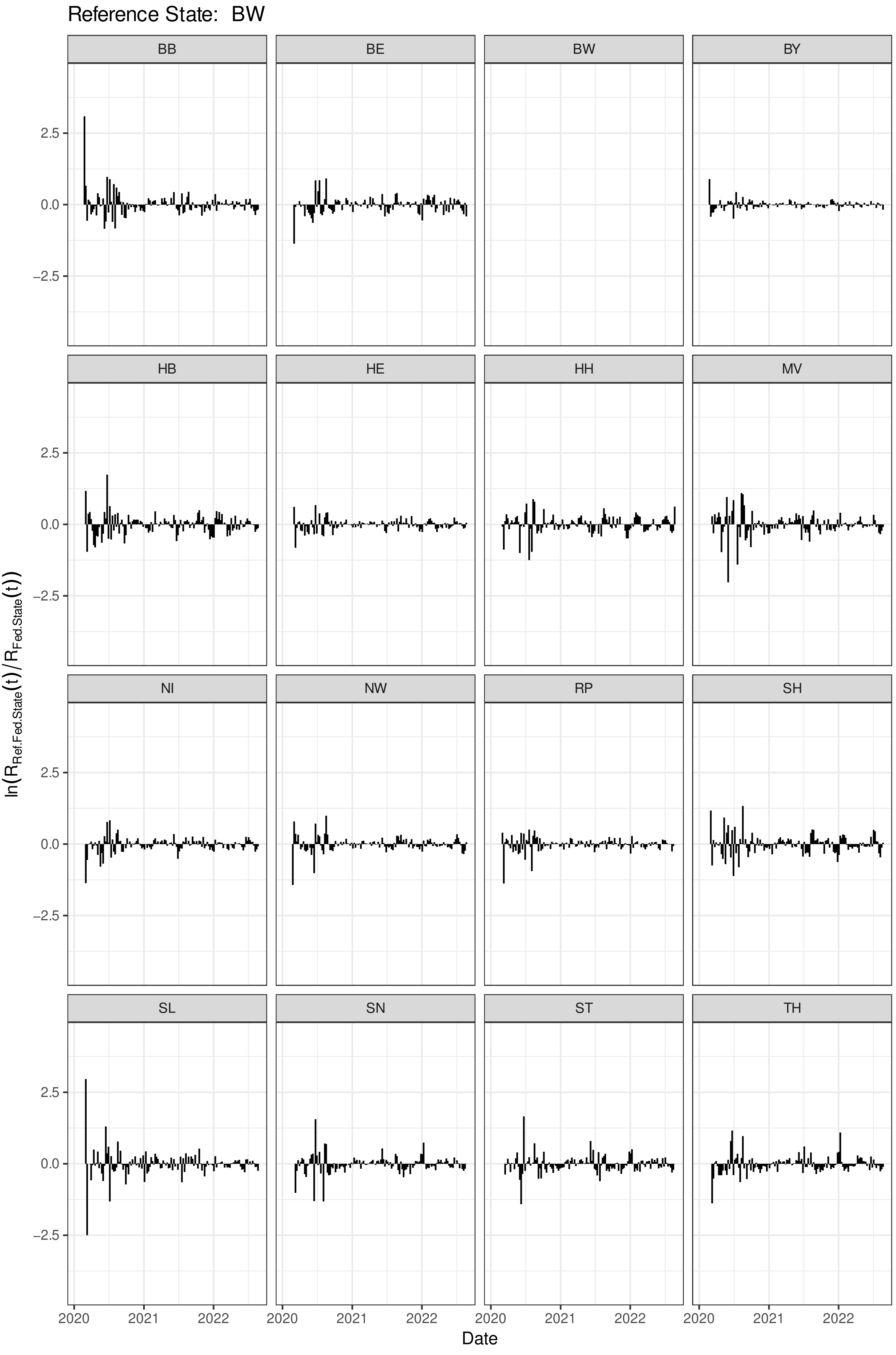

Reference State: BW
BB
BE
BW
BY
HB
HE
HH
MV
NI
NW
RP
SH
SL
SN
ST
TH
2.5
0.0
−2.5
2020
2021
2022
Date
ln(R_Ref.Fed.State(t)/R_Fed.State(t))

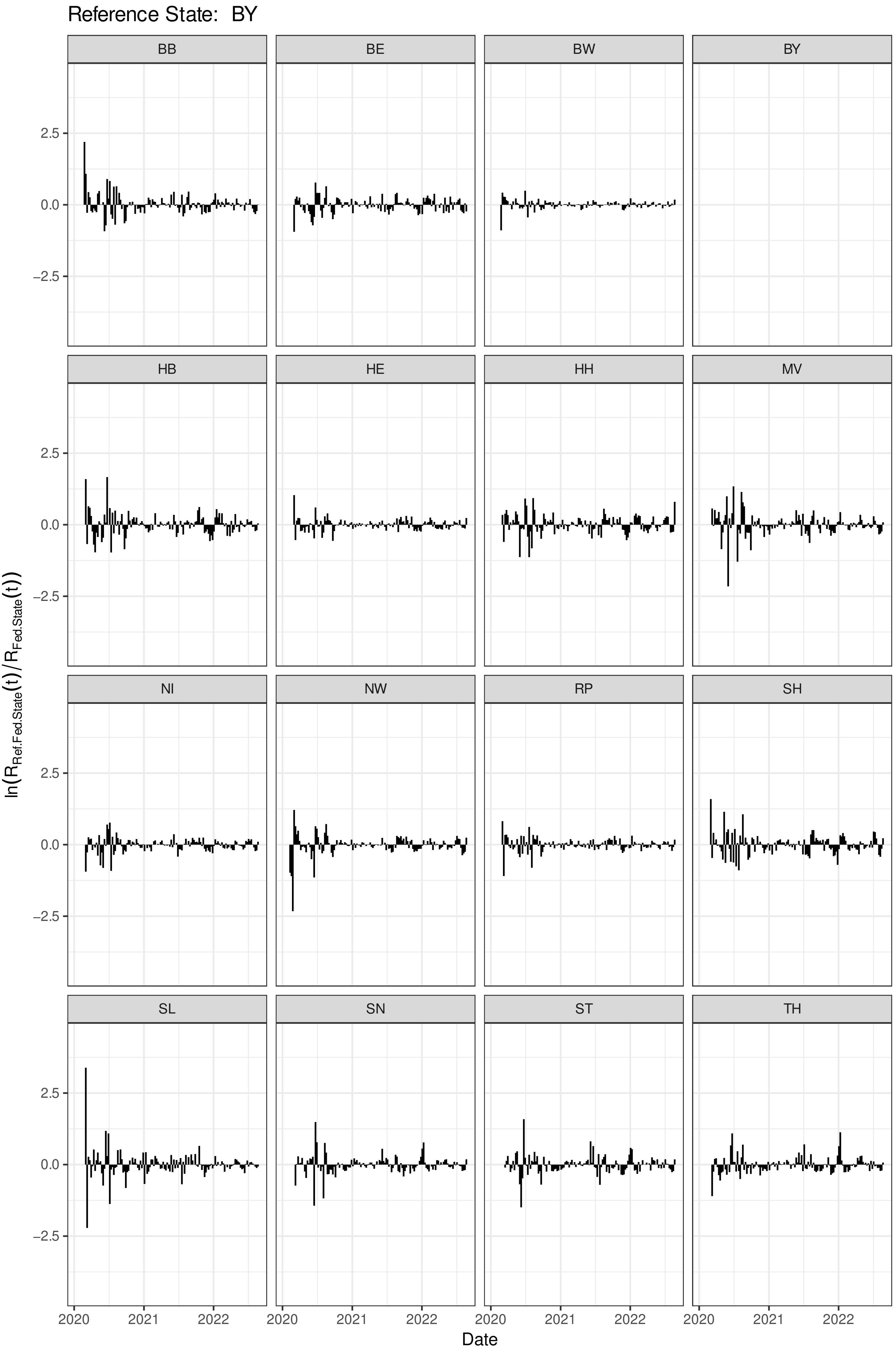

Reference State: BY
BB
BE
BW
BY
HB
HE
HH
MV
NI
NW
RP
SH
SL
SN
ST
TH
2.5
0.0
−2.5
2020
2021
2022
Date
$\ln(R_{Ref.Fed.State}(t)/R_{Fed.State}(t))$

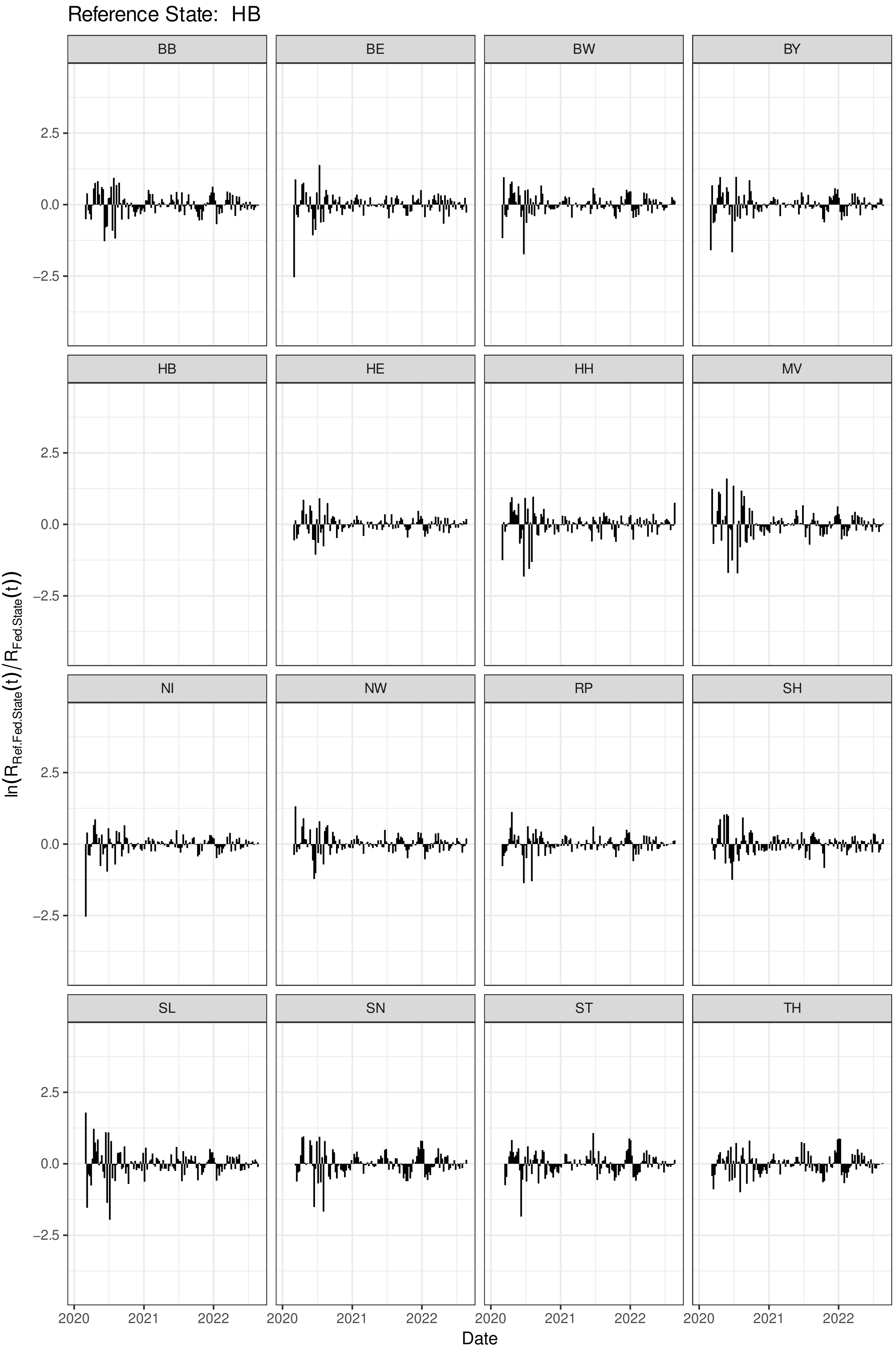
Reference State: HB
BB
BE
BW
BY
HB
HE
HH
MV
NI
NW
RP
SH
SL
SN
ST
TH
2.5
0.0
−2.5
2020
2021
2022
Date
ln(R_Ref.Fed.State(t)/R_Fed.State(t))

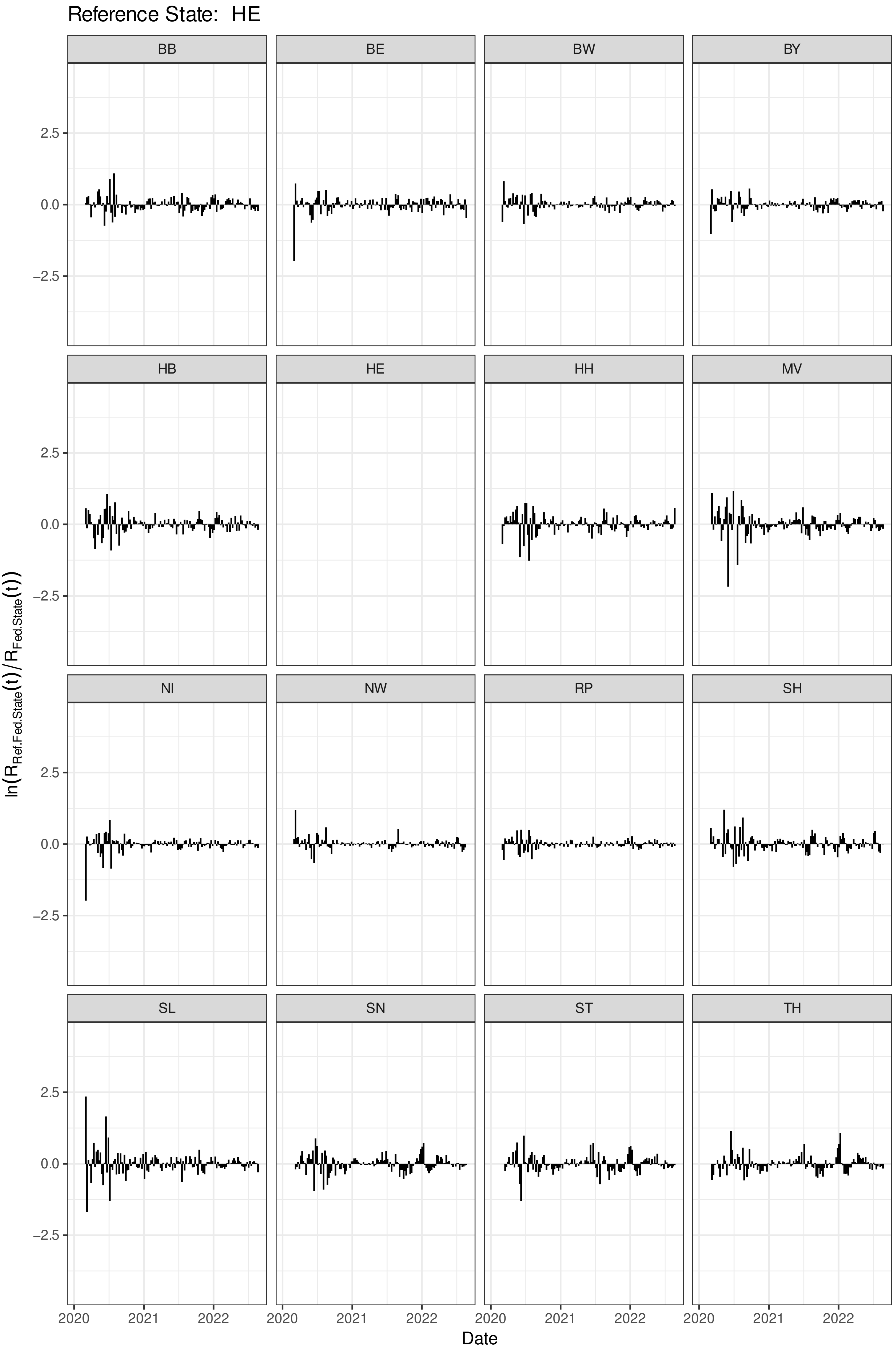
Reference State: HE
BB
BE
BW
BY
HB
HE
HH
MV
NI
NW
RP
SH
SL
SN
ST
TH
2.5
0.0
−2.5
2020
2021
2022
Date
$\ln(R_{Ref.Fed.State}(t)/R_{Fed.State}(t))$

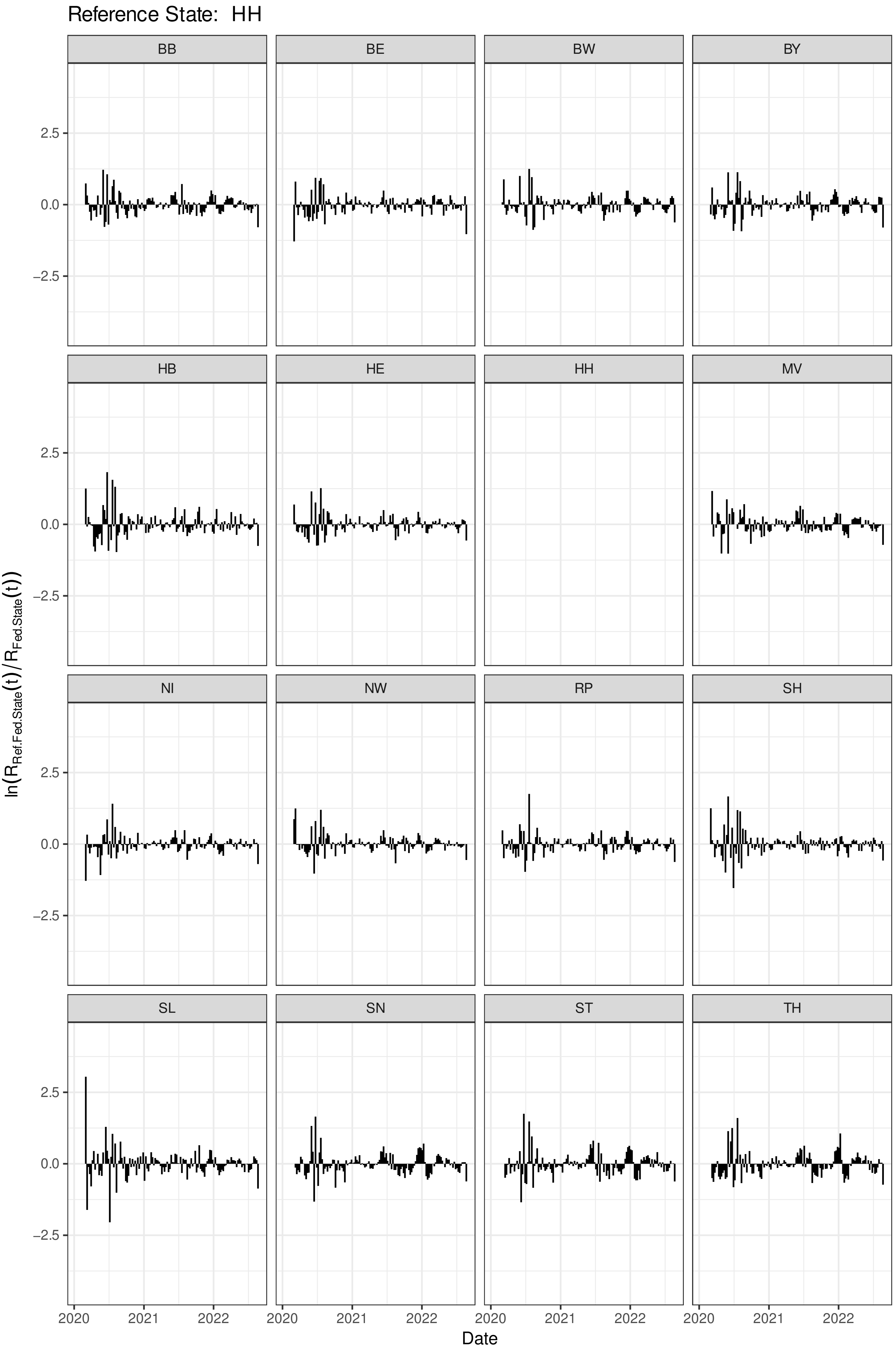

Reference State: HH
BB
BE
BW
BY
HB
HE
HH
MV
NI
NW
RP
SH
SL
SN
ST
TH
2.5
0.0
−2.5
2020
2021
2022
Date
$\ln(R_{Ref.Fed.State}(t)/R_{Fed.State}(t))$

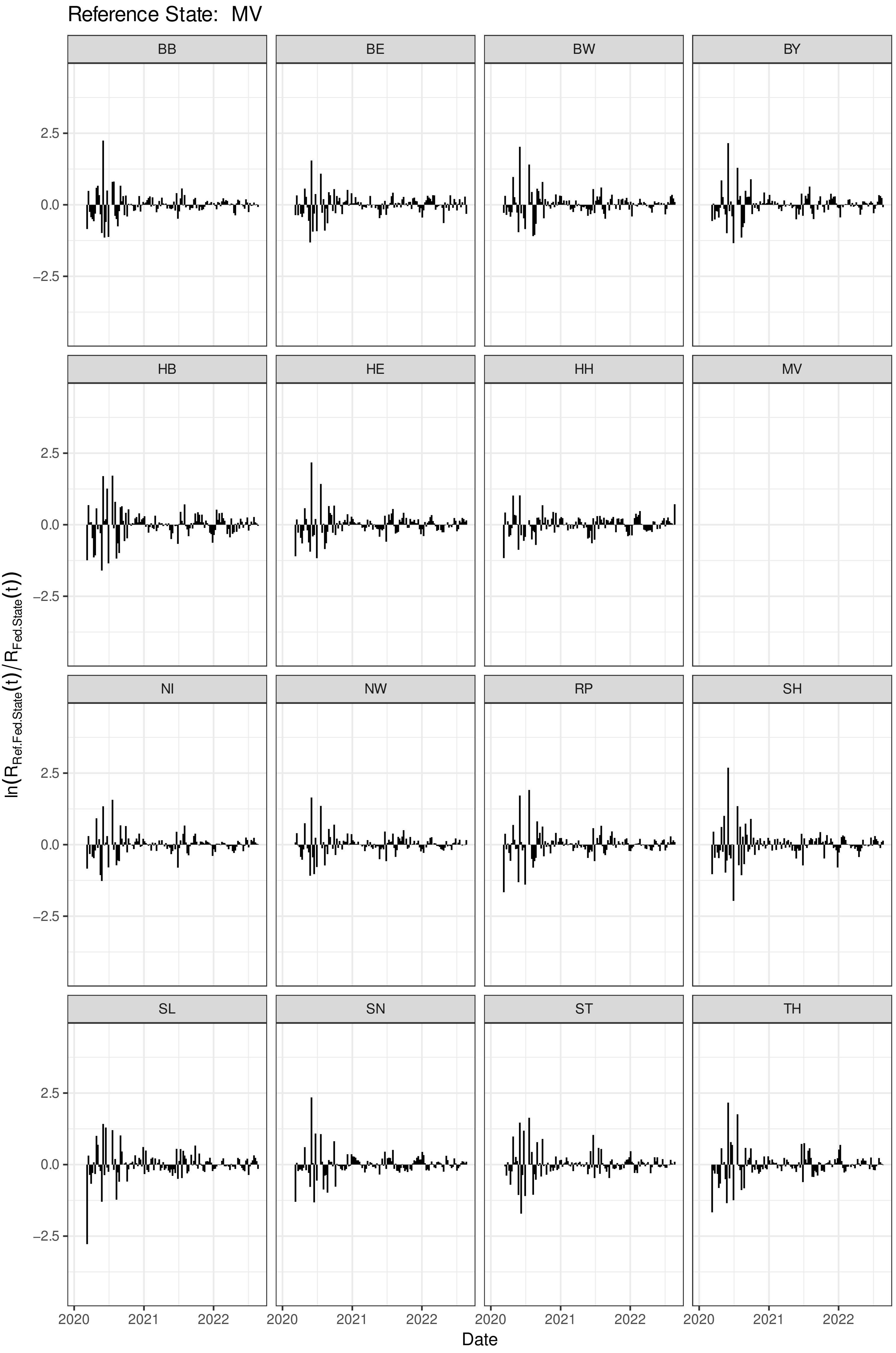

Reference State: MV
BB
BE
BW
BY
HB
HE
HH
MV
NI
NW
RP
SH
SL
SN
ST
TH
2.5
0.0
−2.5
2020
2021
2022
Date
$\ln(R_{Ref.Fed.State}(t)/R_{Fed.State}(t))$

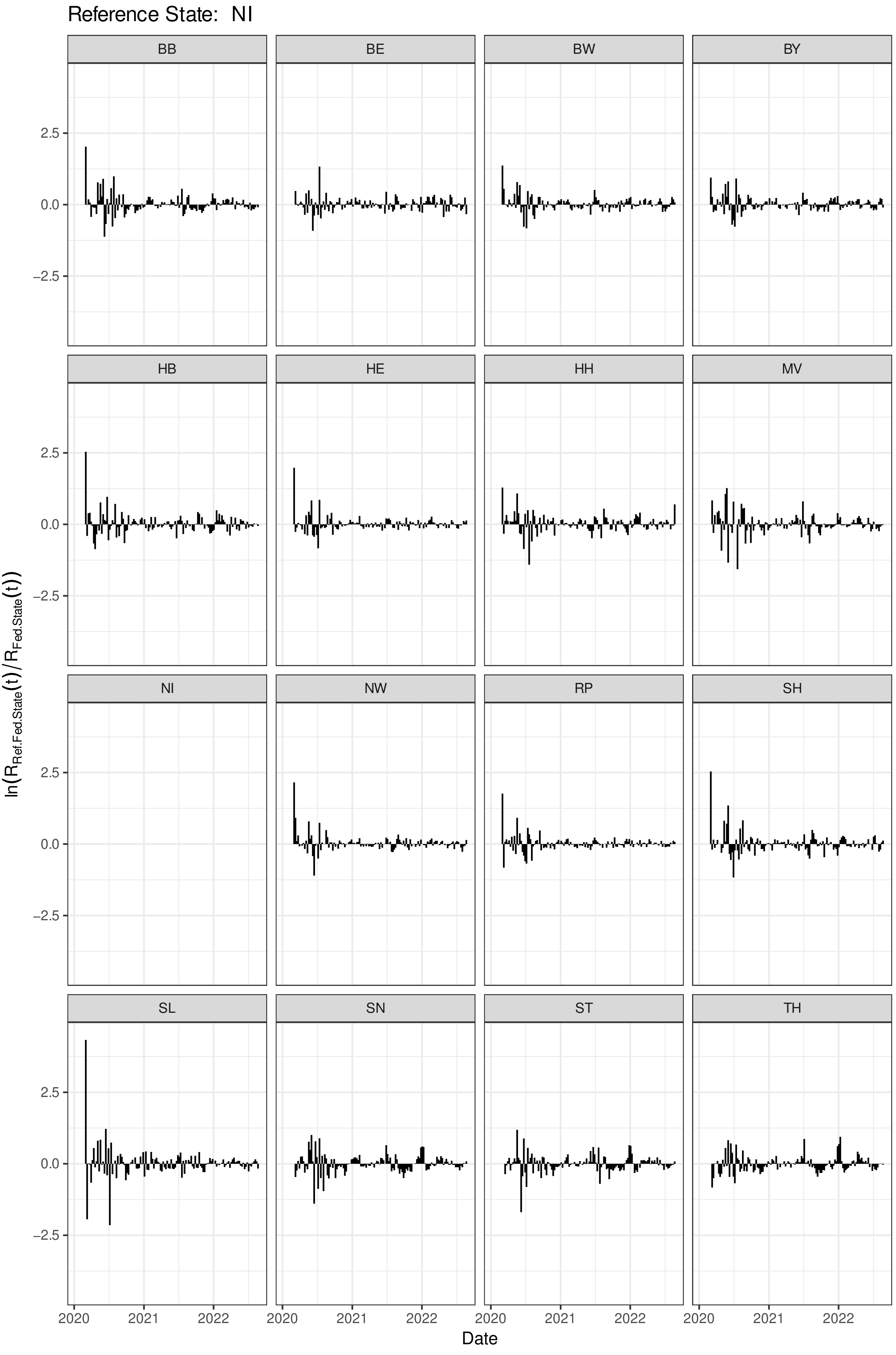

Reference State: NI
BB
BE
BW
BY
HB
HE
HH
MV
NI
NW
RP
SH
SL
SN
ST
TH
2.5
0.0
−2.5
2020
2021
2022
Date
ln(R_Ref.Fed.State(t)/R_Fed.State(t))

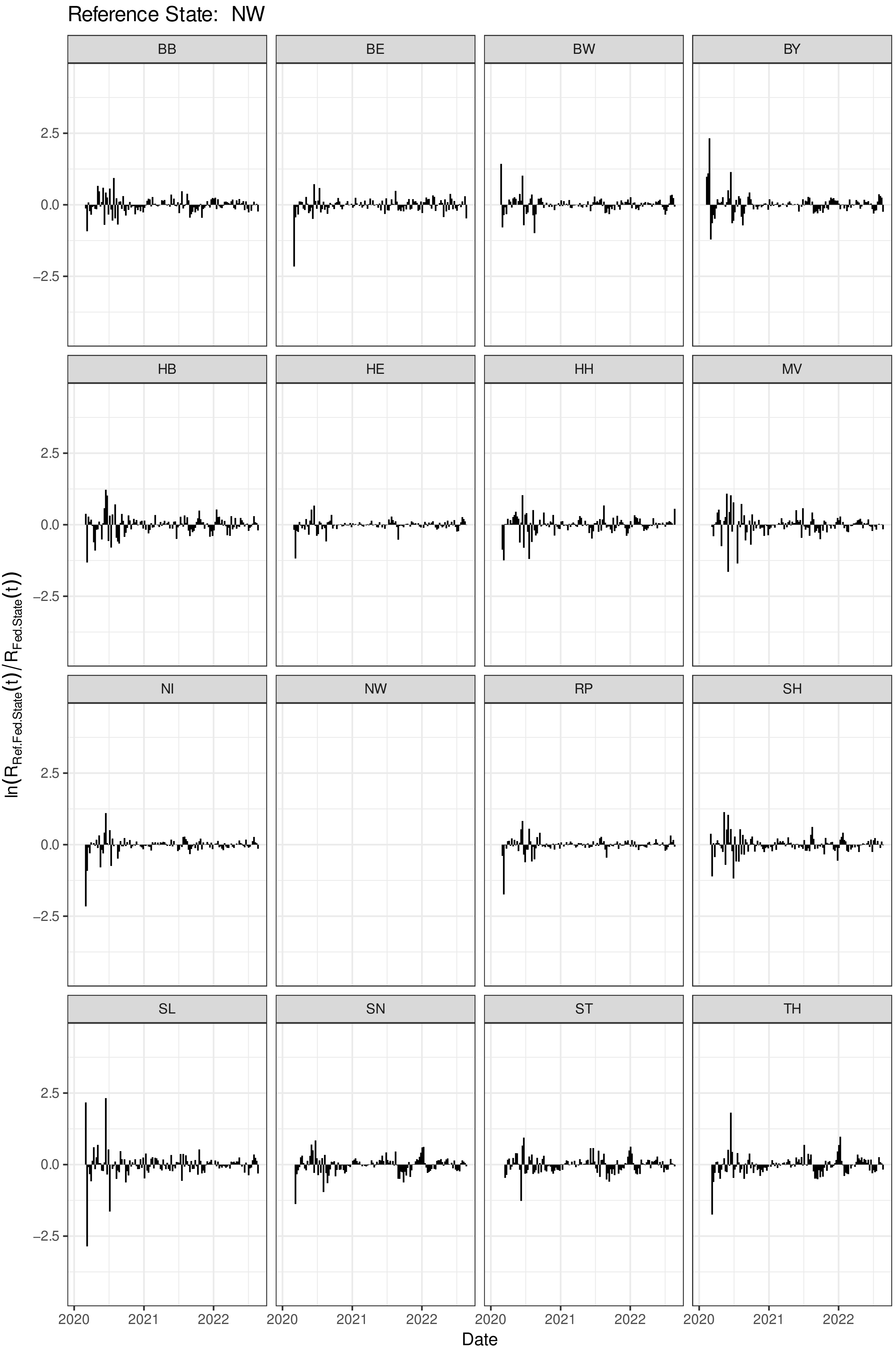

Reference State: NW
BB
BE
BW
BY
HB
HE
HH
MV
NI
NW
RP
SH
SL
SN
ST
TH
2.5
0.0
−2.5
2020
2021
2022
Date
$\ln(R_{Ref.Fed.State}(t)/R_{Fed.State}(t))$

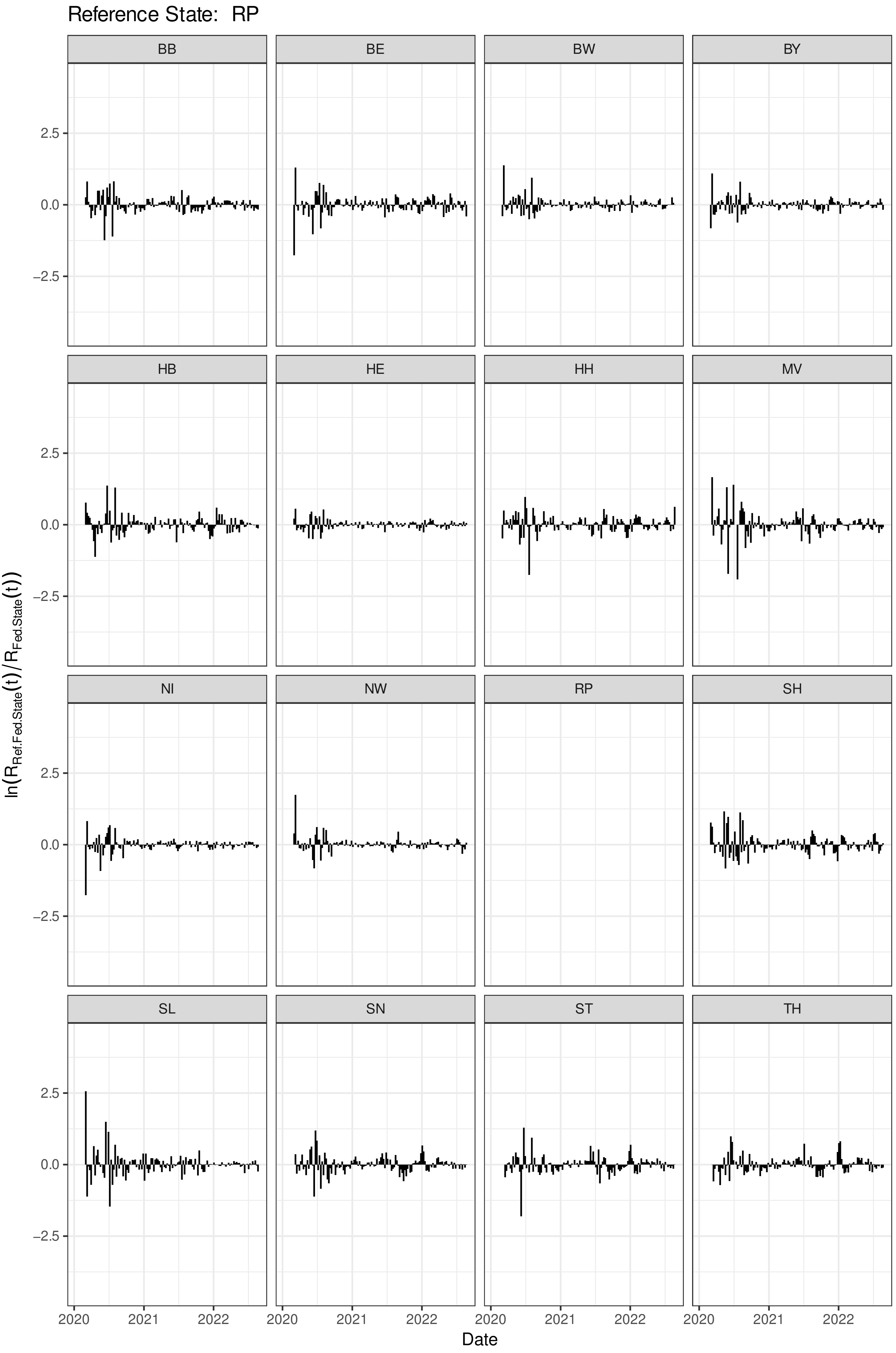
Reference State: RP
BB
BE
BW
BY
HB
HE
HH
MV
NI
NW
RP
SH
SL
SN
ST
TH
2.5
0.0
−2.5
2020
2021
2022
Date
$\ln(R_{Ref.Fed.State}(t)/R_{Fed.State}(t))$

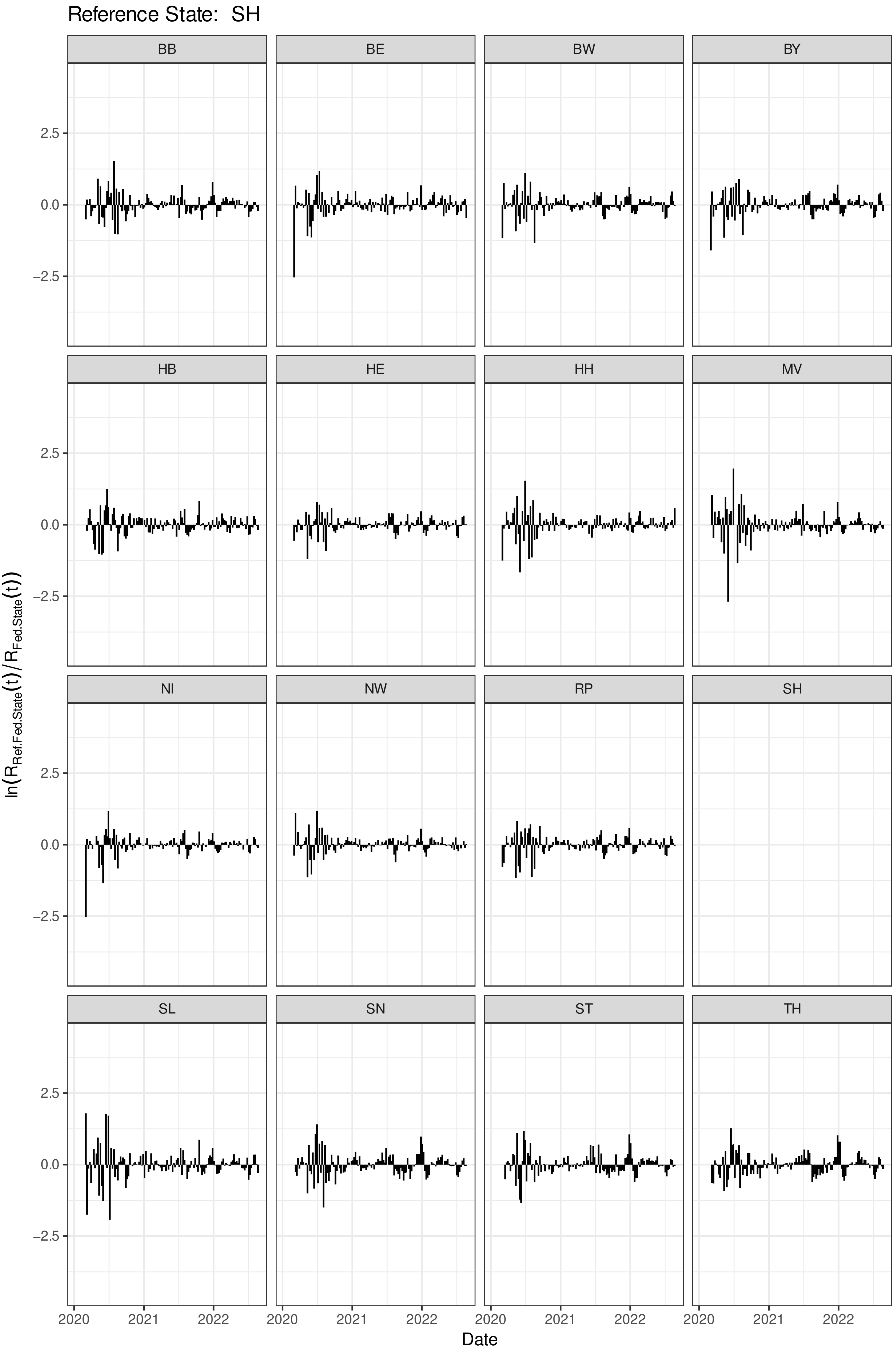
Reference State: SH
BB
BE
BW
BY
HB
HE
HH
MV
NI
NW
RP
SH
SL
SN
ST
TH
2.5
0.0
−2.5
2020
2021
2022
Date
$\ln(R_{Ref.Fed.State}(t)/R_{Fed.State}(t))$

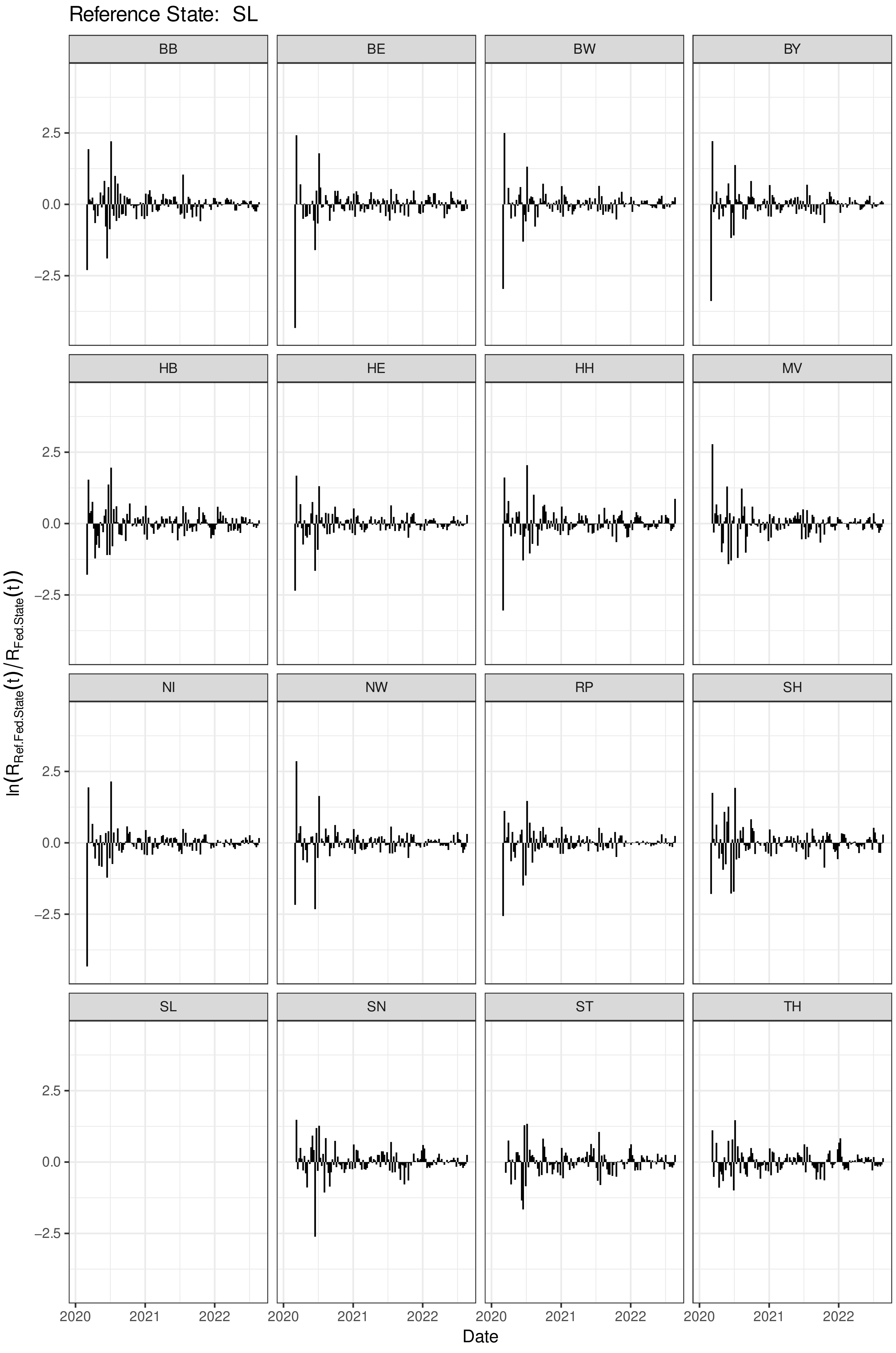

Reference State: SL
BB
BE
BW
BY
HB
HE
HH
MV
NI
NW
RP
SH
SL
SN
ST
TH
2.5
0.0
−2.5
2020
2021
2022
Date
$\ln(R_{Ref.Fed.State}(t)/R_{Fed.State}(t))$

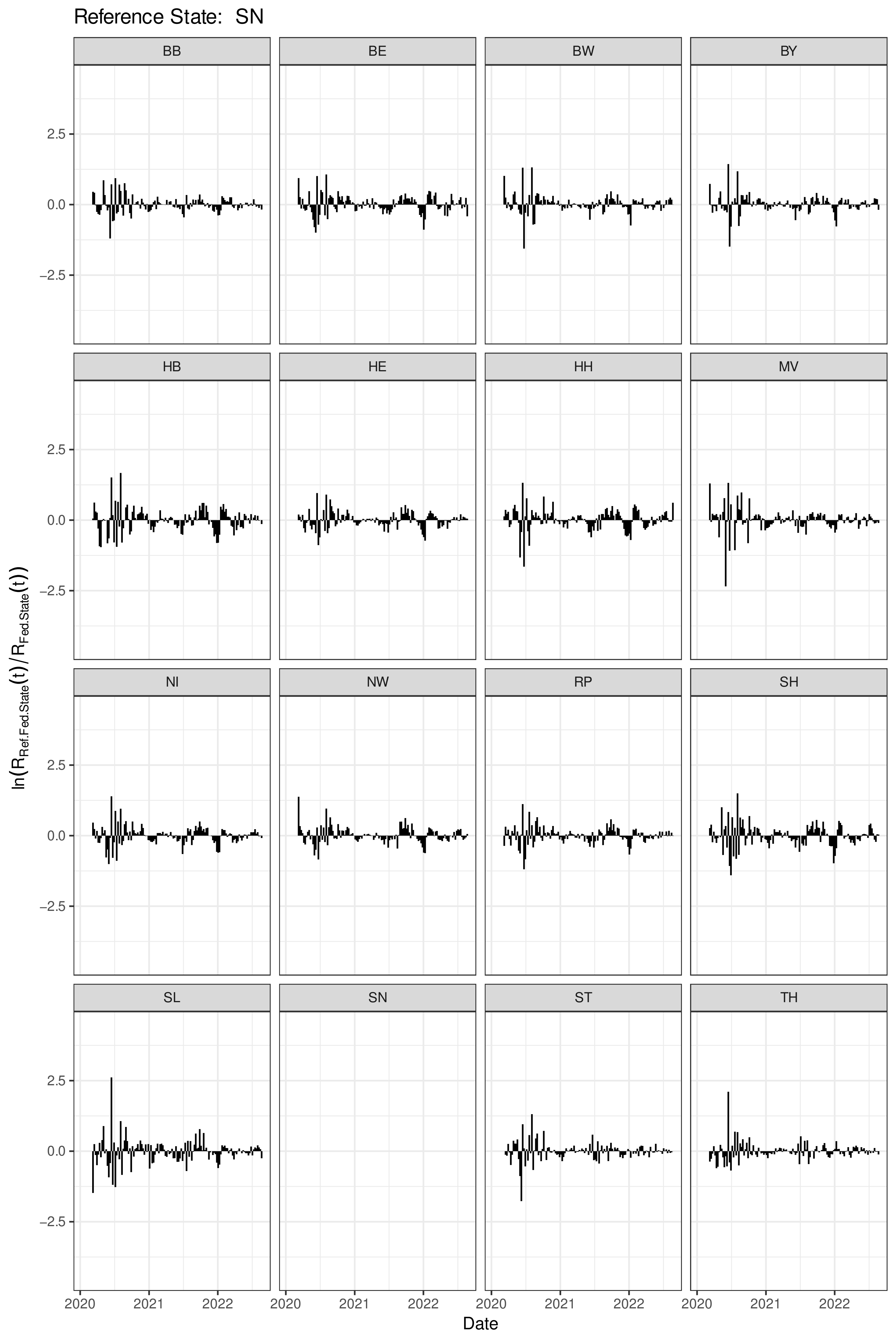

Reference State: SN
BB
BE
BW
BY
HB
HE
HH
MV
NI
NW
RP
SH
SL
SN
ST
TH
2.5
0.0
−2.5
2020
2021
2022
Date
$\ln(R_{Ref.Fed.State}(t)/R_{Fed.State}(t))$

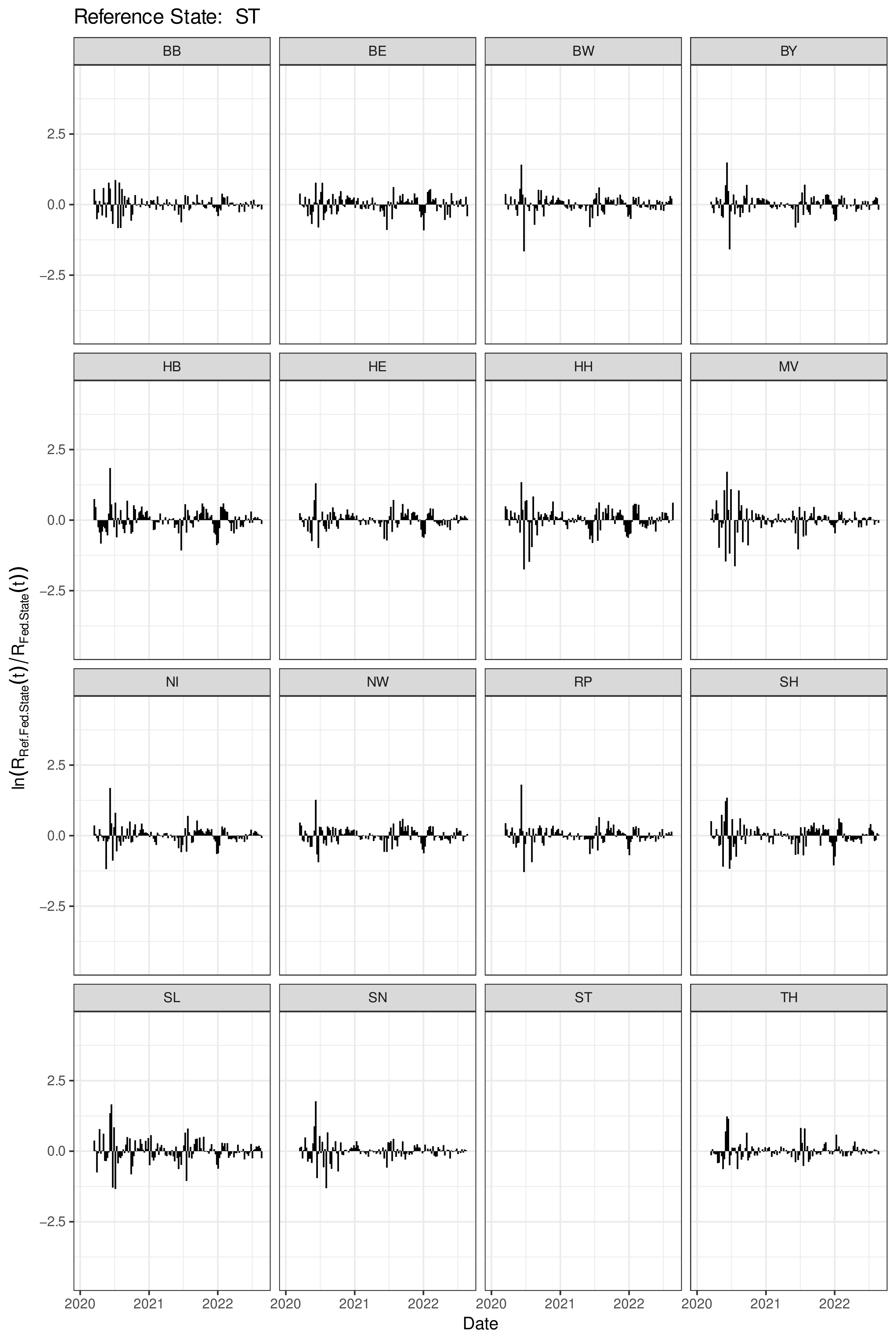
Reference State: ST
BB
BE
BW
BY
HB
HE
HH
MV
NI
NW
RP
SH
SL
SN
ST
TH
2.5
0.0
−2.5
2020
2021
2022
Date
$\ln(R_{Ref.Fed.State}(t)/R_{Fed.State}(t))$

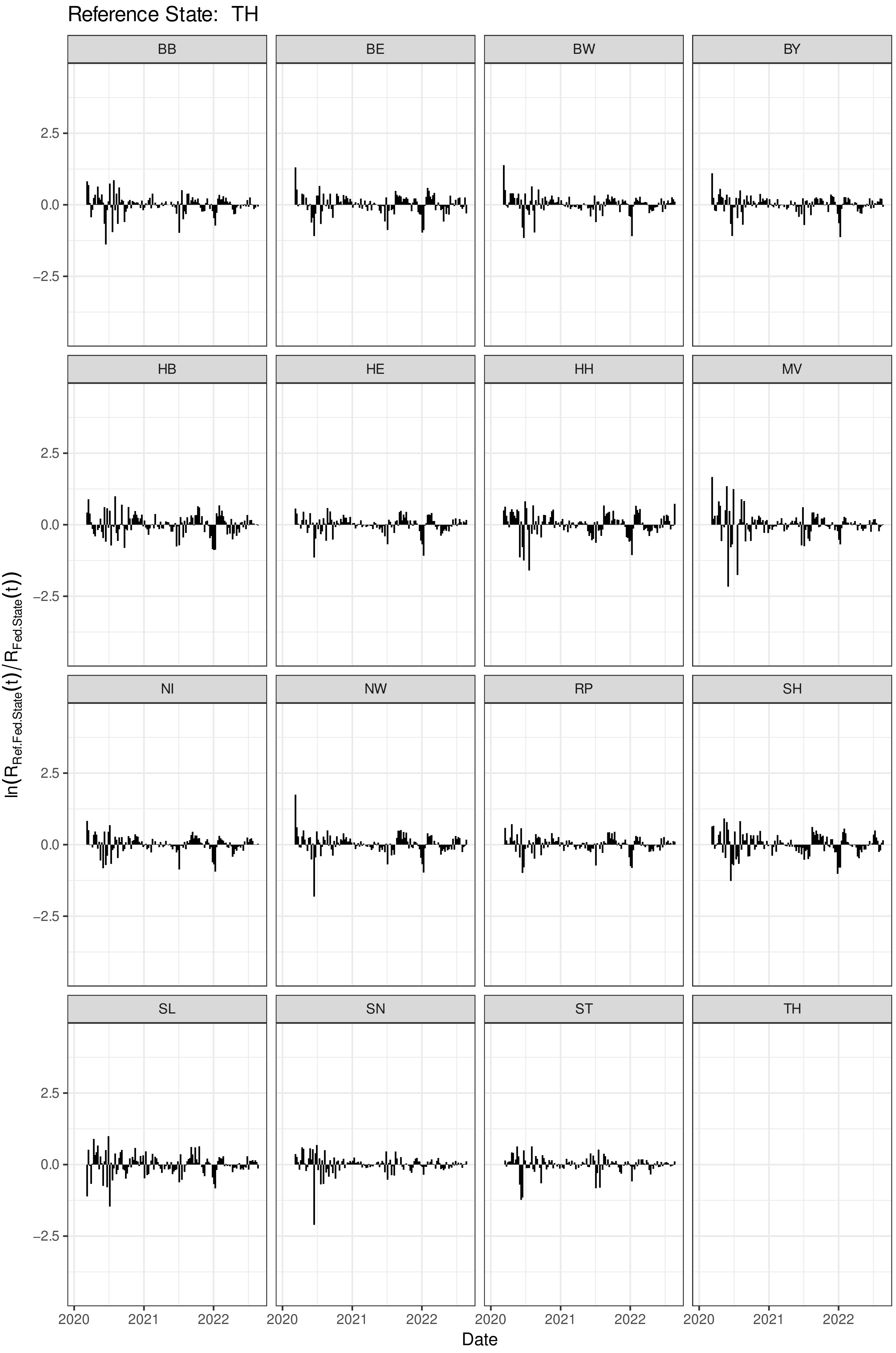
Reference State: TH
BB
BE
BW
BY
HB
HE
HH
MV
NI
NW
RP
SH
SL
SN
ST
TH
2.5
0.0
−2.5
2020
2021
2022
Date
$\ln(R_{Ref.Fed.State}(t)/R_{Fed.State}(t))$
